# Evolving fault diagnosis scheme for unbalanced distribution network using fast normalized cross-correlation technique

**DOI:** 10.1371/journal.pone.0305407

**Published:** 2024-10-17

**Authors:** Balamurali Krishna Ponukumati, Pampa Sinha, Kaushik Paul, Daniel Eutyche Mbadjoun Wapet, Hany S. Hussein, Ammar M. Hassan, Mohamed Metwally Mahmoud

**Affiliations:** 1 School of Electrical Engineering, KIIT University, Bhubaneswar, India; 2 Department of Electrical Engineering, BIT Sindri, Dhanbad, India; 3 National Advanced School of Engineering, Universit´e de Yaound´e I, Yaound´e, Cameroon; 4 Electrical Engineering Department, College of Engineering, King Khalid University, Abha, Saudi Arabia; 5 Electrical Engineering Department, Aswan Faculty of Engineering, Aswan University, Aswan, Egypt; 6 Arab Academy for Science, Technology and Maritime Transport, South Valley Branch, Aswan, Egypt; 7 Department of Electrical Engineering, Faculty of Energy Engineering, Aswan University, Aswan, Egypt; SRM-RI: SRM Institute of Science and Technology (Deemed to be University) Research Kattankulathur, INDIA

## Abstract

There has been a lack of a satisfactory solution for identifying and locating evolving faults in unbalanced distribution systems. The proposed approach is based on the cross-correlation technique as a key element for fault detection and location. Evolving faults, in this context, refer to two sequential faults that result in a change of fault phase. The captured QRS value reflects the occurrence of the second fault occurrence. In order to identify Evolving Faults, it makes use of the signal that is currently being monitored at any given point in the network. Typical system occurrences, such as a short circuit fault that grew into another short circuit fault, as well as cross-country faults, are simulated, and according to the suggested technique, they are accurately differentiated from one another. Using a real-time simulator, rigorous simulations are performed on the modified IEEE 240 bus distribution system. The results of these simulations reveal that they have the potential to uncover defects that are constantly changing. Regardless of the fault (location\resistance\inception angle), location of the monitored point, or sample frequency that is selected, the suggested approach is unaffected by any of these factors. In addition, the slime mold optimization approach is utilized in order to get the best monitoring points that accurately identify the bus in which the evolving fault has taken place.

## 1. Introduction

### a). Motivation and background

Evolving faults (EFs) in a transmission system refer to faulty conditions where the faulted phases change over time. Unlike other types of power system (PS) faults where the faulty phases remain the same and do not change with time, EFs exhibit a dynamic characteristic where the faulted phase evolves or shifts as time passes. The criteria for classifying a fault as an EF are primarily based on the changing faulted phases. EFs pose a challenge in PS protection and require specialized techniques for their detection and location. In order to build protection strategies for PSs in general, a number of different signal processing and mathematical techniques are utilized. For fault detection and protection schemes, a number of signal processing and mathematical techniques are utilized. These techniques include the Stockwell transform (ST), the Wigner distribution function (WDF), the discrete wavelet transform (DWT), the Hilbert transform (HT), the Gabor transform (GT), the Fourier transform (FT), the fast Fourier transform (FFT), and the short time FT (STFT). Currently, the use of these approaches for the purpose of recognizing EFs and defects that span many countries is still in the process of being developed [1, 2].

Understanding and addressing EFs in an 11 kV or 33 kV PS is crucial for ensuring the reliability, safety, and efficient operation of the electrical grid. EFs exhibit dynamic behaviour, with the faulted conditions changing shortly after the fault initiation. This dynamic nature sets them apart from more stable fault conditions. The rapid changes associated with EFs can pose challenges for traditional fault detection methods. Specialized techniques and technologies may be required to effectively detect and analyse EFs. EFs may introduce unexpected challenges, such as unusual fault current levels or other dynamic phenomena. Studying and addressing these challenges is essential for maintaining the stability of the PS. Analysing fault currents associated with EFs is important for understanding the impact on equipment and determining appropriate protective measures. Developing and implementing a robust protection scheme that accounts for EFs is crucial. This may involve the use of advanced relaying techniques and coordination to detect and isolate EFs effectively. Understanding EFs contributes to enhancing the overall resilience of the PS. Identifying and addressing these dynamic fault conditions helps prevent cascading failures and widespread disruptions. Ongoing monitoring and maintenance practices should be adapted to account for the potential presence of EFs. Proactive measures can help prevent equipment damage and mitigate the impact on the PS [3, 4].

The cross-country high impedance fault (CCHIF) can be described as "high impedance ground faults happening in separate phases of one circuit at different places at the same time as the fault inception time" in a typical scenario. Major challenges in CC faults (CCFs) in PSs are the detection of distance, direction, and phase selection in PSs. These faults are simultaneous or EFs in the PS. The relay can fail to work when there is a failure inside its zone or to operate erroneously when there is a fault outside of its zone. This is because the distribution of zero-sequence and negative-sequence components over the protected line, which is employed by the functions that were previously described, is not straightforward to anticipate. This impacts systems that are solidly grounded as well as those that are isolated or impedance grounded [5, 6].

Double line-to-ground faults, simultaneously exist and damage two distinct lines of a given network at two different places, and are frequently referred to as CCFs. The problem is relevant in balanced/unbalanced PS since they experience faults significantly more frequently than HV and EHV transmission networks. CCFs have a particularly negative impact on MV networks with high-impedance grounded, ungrounded, or resonant neutrals since an LGF can result in considerable overvoltage’s on the healthy phases, which can then lead to another LGF and a CCF [7].

### b). Literature analysis

The incorporation of distributed energy resources (DERs) is causing traditional PSs to become more complicated. Both the insertion of DERs into distribution networks and the introduction of the idea of microgrids can assist in meeting the growing demand for electric power. Many key benefits are associated with microgrids, such as the ability to self-heal and self-resilience, enhanced power quality, lower carbon footprints through the utilization of renewable DERs, and efficient operations with reduced line losses. Grid-connected mode (GCM) and islanded mode (IM) are the two distinct modes of operation that microgrids may normally operate in. Microgrids have some challenges when it comes to fault detection. These challenges include variances in short circuit levels between GCM and IM operation, bidirectional power flow, and the minimal fault current contribution of inverter-based DERs among other factors. It is possible that conventional fault detection approaches, such as over-current and directed over-current principles, are erroneous or useless when applied to microgrid settings. The sophisticated fault detection techniques that are required for modern distribution networks (DNs) and microgrids are essential. Many different layouts are utilized by conventional DNs, and the switching frequency can range anywhere from a few weeks to many months. DNs that are reconfigurable entail making changes to the configuration of the network in order to accomplish goals like as lowering losses, decreasing voltage variation, and balancing loads. When DERs that are intermittent, like solar and wind, are present, it may result in numerous hourly reconfigurations. In these kinds of circumstances, the utilization of directional over-current relays necessitates the utilization of several settings, which presents difficulties in terms of obtaining real-time modifications. The integration of DERs brings about many protection concerns, and it brings to light the requirement for sophisticated fault detection techniques. The text refers to a brief overview of protection problems linked to the incorporation of DER in [7], which is not covered in the material that is being presented here. There have been studies conducted on directional overcurrent techniques [8, 9], and the work that is being done in [9] focuses on the coordination of directional overcurrent relays when DERs are present. In [8], the authors suggest the use of new directional components that make use of superimposed impedance to identify both symmetrical and asymmetrical defects. There have been attempts made to develop adaptive protection mechanisms [10, 11].

Based on the findings of the study [10], an adaptive settings-based protection system that is dependent on communication infrastructure is proposed. It is claimed in [11] that a superimposed current-based direction estimation can be used to improve the functioning of overcurrent relays. This estimation makes use of the angle difference between the pre-fault current and the superimposed current to determine the direction of the fault. An adaptive settings-based distance relay is proposed for active DNs, which is one of the distance protection solutions that have been investigated [12]. On the other hand, this technique is not investigated with active sources that are dependent on inverters.

Learning-based defect detection techniques that make use of neural networks, support vector machines (SVMs), decision trees, and deep learning have been the subject of research [13–15]. Many differential characteristics are utilized for protection in the work described in [13], which asserts that symmetrical components of current are the most effective features for fault detection. There are techniques for protecting microgrids that are based on data mining that are presented in [14]. Ref. [15] proposed a protection method for microgrids that makes use of WT and deep learning. This scheme offers information on the fault, including its location and the sorts of faults that it can experience. In [16], a differential protection system that is based on instantaneous current is suggested. This system checks the difference in current for two samples that are taken consecutively. On the other hand, this method is only useful in situations when the fault current is at least ten percent higher than the nominal value.

Due to the substantial quantity of data that is required, verification of learning-based protection methods continues to be a difficult task. These studies shed light on the many ways that academics have tried to handle the issues of fault detection and protection in microgrids and DNs that are constantly developing over time. Microgrids are becoming increasingly utilized in DNs to develop applications that involve monitoring, protection, automation, and control activities. The availability of low-cost hardware platforms that are capable of achieving protection criteria [17] and effective synchro phasor estimate algorithms are the factors that have contributed to the implementation of this technology becoming feasible. Synchro phasor data-based state estimators were used in earlier studies [18, 19] for fault identification through the procedure of estimating measurement residuals. These systems, on the other hand, demonstrate a high level of numerical complexity and reaction times. Parallel weighted least square state estimation and zero sequence current are the foundations of yet another fault detection approach [20]. In GCM, it is confirmed even though it takes into account a variety of network topologies. However, it does not analyze to determine the variance in fault resistance. According to the technique presented in [21], a fault localization method was proposed that involves calculating the injection fault current by utilizing the impedance matrix. On the other hand, it draws attention to a problem in which many configurations can have the same impedance matrix, which would result in inaccurate fault identification and placement. A further approach for fault detection that is data-driven [22] uses an SVM to differentiate between events. On the other hand, it is mentioned that data-driven algorithms need a significant quantity of fault and no-fault event data and that the selection of a hyperplane for the SVM could be difficult. The challenges in the discussed methods include the numerical complexity and high response time of state estimation schemes, potential issues with fault resistance variation in certain methods, and the requirement for a significant amount of data for data-driven algorithms.

DNs are more extensive and intricate due to the presence of various loads, transformers, and branching. EF detection techniques designed for transmission lines may not account for the complexity and diversity of components in PSs. Fault characteristics in DNs may differ from those in transmission lines. Distribution systems often experience more diverse fault types, including single-phase and two-phase faults, which may require different detection approaches. Availability and quality of data for fault detection play a crucial role. Transmission systems may have more robust monitoring and measurement infrastructure, whereas DNs may have limitations in terms of sensor density and data granularity. DNs may have cost constraints that limit the deployment of sophisticated monitoring and detection technologies. This can affect the feasibility of implementing EF detection techniques that may require additional investments [23].

### c). Significant contributions

A cross-correlation-based method for the EF detection scheme is suggested in the current work. A safeguard system should be created to identify EFs, which could cause equipment damage or prolonged outages if not identified. Conventional and numerical relays were not effective enough for the detection/classification of EFs in the case of CCFs syndrome on a widely dispersed, complicated power system network, which is where the problem has become most severe in recent years. CCFs, which impact numerous sites concurrently and multiple phases in large imbalanced DNs, have not previously been studied. In light of this restriction, the essay focuses solely on an imbalanced 240-bus distribution network topology, exploring its various conceivable configurations. Justification of the proposed method has been reported in Table 1.

**Table 1:** Q, R, and S values for different disturbances.

Type of Fault	Q	R	S
	0 to 90°	0 to 90°	0 to 90°
EFs	20 to 110	20 to 100	20 to 90
CCFs	2 to 11	2 to 10	2 to 9
short circuit fault	0.2 to 0.11	0.2 to 0.1	0.2 to 0.9

The innovative contributions of the proposed methods are as follows:

In light of the findings of this research, we propose a method that is based on CC for locating faults in DNs that come from international EFs. To determine the signature qualities, the technique takes into account the cross-correlogram by comparing the voltage during the healthy condition to the voltage during the defective state.

It is demonstrated that the correlogram can lessen the impact of uncorrelated random noise, which validated its usefulness as a tool for data analysis. It is the goal of feature extraction to produce new features from current features in a dataset, which will ultimately result in the dataset being smaller in size. The new features, which have been simplified, should be able to properly summarize the amount of data that is contained in the previous features. Creating a more simplified version of the original set is possible through the process of integrating the original elements in this manner.

One approach that is effective in lowering harmonics is the sparse nonnegative matrix factorization (SSNMF) algorithm, which can automatically extract sparse and relevant features from a given set of nonnegative data vectors. An approach validated on a real-time platform. In this study, we introduce a technique for identifying EFs via CC. We next go over how to determine where exactly a monitoring point needs to be set up, after which we briefly discuss the correlation method and the method of peak detection (MOPD).

## 2. Proposed method

### a). Feature extraction based on time cross-correlation

A statistical method known as correlation describes how closely two variables are related. Correlation is helpful because it shows the connection between two variables, which enables us to forecast how the system will behave in the future. Correlations can be either autocorrelative or cross-correlative. The random noise and uncorrelated noise that are present in the signals are not reflected in the cross correlogram of the two signals [24, 25], which does not reveal the influence of these effects. A time series is defined as a set of triplets (ti, fi, ei), where ti is the time of the observation, fi is the measured value of the quantity of interest (such as the flux density or photon flux), and ei is an estimate of the observational error associated with the measurement. Specifically, we expect the time series to be sorted in order of time, with I = 1, …, N. The periodogram is traditionally defined as the squared modulus of the discrete FT (DFT), and it can be used to approximate the power spectral density (PSD) [26]:

$$P(v_{k})=\frac{2T}{N^{2}}\left({\left[\sum_{i=1}^{N}f_{i}\cos(2\pi v_{k}t_{i})\right]}^{2}+{\left[\sum_{i=1}^{N}f_{i}\sin(2\pi v_{k}t_{i})\right]}^{2}\right)$$ (1. a)

By applying normalization to the CC function, we can obtain a Pearson correlation coefficient that is time-adjusted.


$$\rho xx(t_{1},t_{2})=\frac{K_{xx}(t_{1},t_{2})}{\sigma x(t_{1})\sigma x(t_{2})}=\frac{E\left[(X_{t_{1}}-\mu_{t_{1}})\bar{\left(X_{t_{2}}-\mu_{t_{2}}\right)}\right]}{\sigma x(t_{1})\sigma x(t_{2})}$$ (1. b)

Specifically, a stochastic process’s normalized cross-correlation is defined as

$$\rho xy(\tau )=\frac{K_{xy}(\tau )}{\sigma x\sigma y}=\frac{E\left[(X_{t}-\mu x)\bar{\left(Y_{t+\tau }-\mu y\right)}\right]}{\sigma x\sigma y}$$ (1. c)

$$=\text{K}xy(\tau )=E[x(t1)y(t1+\tau )]=E[y(t_{1})x(t_{1}-\tau )]=\text{K}yx(-\tau )$$ (1. d)

where μ_x_ and σ_y_ are the mean and standard deviation of the process (X_t_) which are constant over time and similarly Y_t_ and K_xy_ are cross co-variance functions respectively.

In this case, the frequencies are given by v_k_ = k/T, where k can be any integer from 1 to N, and v_k_ = k/(N 1)/2, where N is an odd number. The minimum frequency is ν_min_ = 1/T, the maximum frequency is the Nyquist frequency ν_Nyq_ = (N/2)(1/T) and T = N(t_N_ − t_1_)/(N − 1).

The CC between the two signals, x(n) (Reference signal) and y(n) is presented below for your perusal.

$${\hat{R}}_{xy}(m)=\left\{\begin{array}{c} \sum_{n=0}^{N-m-1}x_{n+m}y_{n}\\ {\hat{R}}_{yx}(-m)\; \end{array}\right.\left.\begin{array}{c} m\geq 0\\ m<0 \end{array}\right\}$$ (2)

where m is equal to… 2, 1, 0, 1, 2,… Subscript xy denotes the order in which the two variables are associated, and index m denotes a parameter that shifts with time. One sequence is shifted about another sequence, and the order of the subscripts, with x appearing before y, specifies the direction in which the shift occurs. This is in stark contrast to the scenario for unequal sampling, where the substantial red-noise leakage observed in the simulations and the increased noise can be attributed to the morphologies of the window functions. Using even sampling, we can re-create the standard Fourier analysis results, complete with the well-established characteristics of window functions.

In case of representing noise then we can write

x(t)=Acos(ωt+θ) (3)

where θ is the random variable and y(t) represents noise. *x*(*t*) & *y*(*t*) are uncorrelated functions. Then autocorrelation coefficient of x(t) is $Rx(\tau )=\frac{A^{2}}{2}\mathrm{Cos}\omega \tau$. *Ry*(τ) is an autocorrelation function of noise y(t). *Ry*(τ) should be decaying in nature, Hence correlograms are decaying in nature which are shown in Fig 10.


$$Ry(\tau )=Y_{0}^{2}e^{-\infty /\tau }$$ (4)

After adding x(t) and y(t) we get,

z(t)=x(t)+y(t) (5)

Z = *A cos*(*ωt*+θ)+*noise*

$$R_{z}(\tau )=Rx(z)+Ry(\tau )\;Y_{0}^{2}>>\frac{A^{2}}{z}$$ (6)

If x(t) contains several frequencies or a small band of frequency where this band of component can be recovered from the new signal Z(t). In this research non-negative matrices SSNMF algorithm has been used to remove the significant noise.

### b). Reducing of noise

The effectiveness of the technique for source separation using non-negative matrices SSNMF has been demonstrated in [27]. One way to graphically portray the SSNMF approach is by the use of a sweep series of amplitude spectra. As a result of the SSNMF decomposition, the frequency-following response (FFR) can now be observed with more clarity, and any additional noise that was present in the recordings has been removed. An exponential curve fit is used to develop a model, and the patterns of FFR improvement and noise reduction with an increasing number of sweeps are investigated. The model is then generated. Among the potential applications of the SSNMF method on the FFR signal is the evaluation of pitch processing and neuroplasticity processes in the electrical signal when it is subjected to disruptions [27]. The letter k in this notation denotes the kth basis out of a total of n bases, while the letters I and j stand for elements along the first dimension of a matrix, which is a flattened vector of frequency time, and the second dimension, which is a sequence of amplitude spectrograms, respectively. Applications of the NMF technique demonstrated that the matrices W and H were able to acquire the part-based representation that was required to reconstruct the data. This was because V and H did not have a negative value. Because of this constraint, the notion that the incoming data was a linear sum of numerous sources was reinforced, which reflected the additive nature of electrical impulses.

To initialize the matrices W and H, random values were utilized, and then the standard NMF multiplicative update procedure was applied to them. The effectiveness of the SSNMF algorithm is demonstrated by the sweep series of amplitude spectrograms [27], which are derived from recordings of both adults and newborns. Due to the fact that this is the situation, the remedy that has been suggested makes use of this tactic.

Machine learning algorithm that takes a non-negative input matrix (A) and uses it to learn and factorize another, smaller matrix (S) that serves as the spectral basis and (T) that serves as the information coding.


$$A_{ij}\approx {\left(ST\right)}_{ij}=\sum_{k=1}^{n}S_{ik}T_{kj}$$ (7)

**Fig 1:**
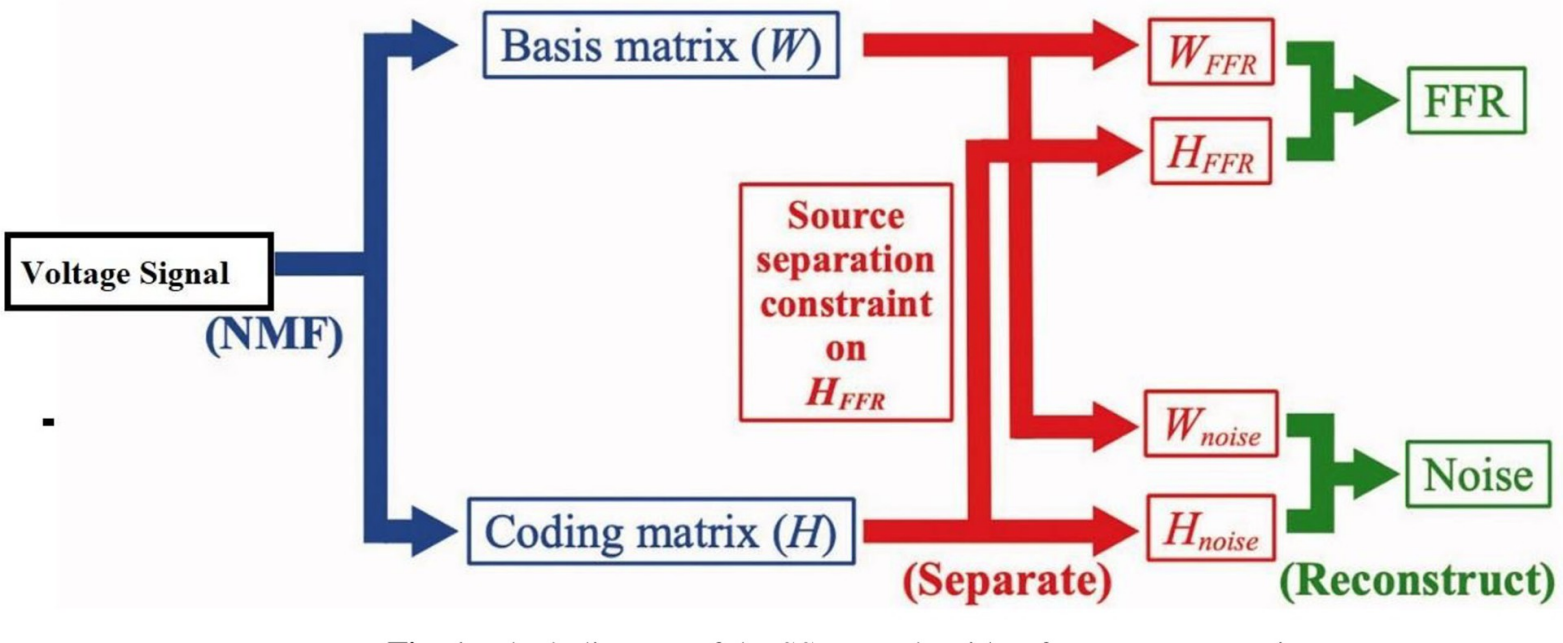
Block diagram of the SSNMF algorithm for source separation.


I and j were used to denote elements along the first dimension of the A matrix, which was a flattened vector of frequency time, and the second dimension, which was a sequence of amplitude spectrograms, respectively. The letter k was used to indicate the k-th basis out of a total of n bases. A total of n dimensions were present in the matrix. A comparison of the amplitude spectrograms that were created is the reason why this is done. These two bases, which together make up the spectral-basis matrix S, were required to learn and differentiate between the spectral features of the FFR and noise, respectively, as a result of this limitation. Once this method was implemented, the FFR and the noise were independently recreated, as seen in Fig 1. The formulas that were utilized for this reconstruction are as follows: The input data matrix T is multiplied by the S-T ratios of each of the elements, and the resulting product is then divided by the matrix T.

$$FFR=A{}^{\circ}\left(S_{FFR}T_{FFR}\right)/ST$$ (8)

$$Noise=A{}^{\circ}\left(S_{noise}T_{noise}\right)/ST$$ (9)

Eq (10) is used to outline the improvement of efficiency in the FFR signal (i.e., performance of the SSNMF method).

$$B(n)=B_{BS}e^{\left(-\frac{n}{\tau }\right)}+B_{DC}$$ (10)

where B was the performance index (FFR Enhancement), BBS was the asymptotic amplitude of the fitted curve without the direct current component, n was the number of sweeps in each signal, e was Euler’s mathematical constant = 2.7182, which showed the fitted curve’s time constant (that is, the number of sweeps needed to reach 63% of the asymptotic amplitude), and BDC was the fitted curve’s direct current component. For Noise Reduction, a different model was used, and the results showed that as the number of sweeps went up, they got better: $B(n)=B_{BS}e^{\left(-\;\frac{n}{\tau }\right)}+B_{DC}$

$$FFR\;Enhancement=0.254*(e^{\left(-\frac{n}{555}\right)})+0.005$$ (11)

$$Noise\;Reduction\;form\;signal=20.653*(1-e^{\left(-\frac{n}{290}\right)})-20.991$$ (12)

### c). Method of peak detection (MOPD) for determining QRS values

Using normal-exponential-Bernoulli (NEB) and mixture probability models, we have applied a brand-new peak identification algorithm [25], for the analysis of extensive two-dimensional electrical signals.

$$X_{i}∼ND\left(\theta_{i}+\mu ,\;\sigma^{2}\right)\;and\;\theta_{i}∼Exp\left(\phi \right)$$ (13)

where ND represents a normal distribution and Xi represents a TIC that has been detected To be more specific, a chromatogram is referred to as a TIC when it is created by combining the intensities of all of the mass spectral peaks that are collected in a single scan.

To restate, we pretend that the noise has a mean of zero and a variance that is distributed according to the normal distribution. At the ith point, the real TIC of Xi is represented by θi, which is the exponential distribution with φ. Additionally, μ represents the mean backdrop or baseline with a deviation of σ2. Measurements from sensors have the potential to considerably improve one’s comprehension of the behavior of complicated systems. To improve decision-making in system management, the quality of the data collected by sensors is very essential. The selection of monitoring systems, which may include the types of sensors and the configurations of those sensors, is frequently determined solely by engineering judgment. As a result of the relatively low cost of sensor devices, huge sensor networks are deployed to gather data at high frequencies over extended periods. This results in the collection of very large datasets; nevertheless, model predictions of system behavior frequently rely on only a few factors. There is a possibility that informative data will be buried by data that is redundant or irrelevant when critical parameter values are being updated. Within the scope of this study is a system for picking the most appropriate measuring locations.

### d). Optimal choice of monitoring points based on slime mold algorithm (SMA)

Installation of smart meters (SMs) and the identification of the fault in the distribution line are both going to be necessary to determine the nature of the issue that is occurring. If fewer SMs are installed, it will be able to reduce the overall cost of the installation without sacrificing efficiency. It is necessary to put into action an essential optimization approach to reduce the number of SMs and zero in on the location that would be most appropriate for one of these devices. To determine the most suitable location for the SM, this work makes use of an optimizer that is founded on the SMA. It is generally agreed upon that the objective function of the optimal placement problem (OPP) should be understood to be [28]:

$$\text{Minimize}\sum_{k=1}^{n}Z_{k}$$ (14)

Subjected to $\left[C\right]*\left[Z\right]\;\geq \;\left[b\right]$

C represents a connection matrix, and n represents the total number of buses. This is the representation of the Matrix C, which is as follows:

$$Matrix\;C_{ij}=\left\{\begin{array}{c} 1,\;if\;i=j\\ 1,\;if\;i\;and\;j\;are\;connected\\ 0,\;if\;other\;wise \end{array}\right.$$ (15)

whereas B is a column matrix and it is represented as $[b]={\left[1\;1\;1\;1\;1\ldots ‥\;1\right]}_{1XN}^{T}$

### e). Applied SMA

A behavioral characteristic of SM served as the inspiration for the SMA, which was proposed by Li Shimin and colleagues [29]. The SM is responsible for identifying the presence of food in the natural environment, following which it encircles the food and finally utilizes enzymes to digest it. The characteristics of SM may be mathematically defined as three processes: searching for food, encapsulating food, and oscillating. These three steps can be represented as follows from a mathematical perspective. The slime mold tracks the food based on the smell dissipated in the air which can be represented as [30]:

$$Z\left(k+1\right)=\left\{\begin{array}{c} Z_{b}(k)+vb.(H.Z_{A}(t)-Z_{B}(t)),\;r<p\\ vc.Z(t),\;r\geq p \end{array}\right.$$ (16)

In (16), Z is the SM’s position, Zb is the most recent location with the strongest smell (the food location), ZA and ZB are SM candidates chosen at random, r is a random integer between 0 and 1, k is the number of iterations, H is the adaptive weight of the SM, vb is a randomly generated value between -a and a, and vc is a randomly generated value between -b and b, where b is a value that decreases linearly from 1 to 0 depending on the iteration (b = 1-k/Itermax). One way to express the probability index p is in (17):

p=tan|J(i)−EG| (17)

Here in (11), *J(k)* represents the fitness value corresponding to *Z*, and *EG* resembles the best candidate solution achieved so far. The parameter *a* can be represented as:

$$a=\arctan\;h\left[-\left(\frac{k}{Iter_{\max}}\right)+1\right]$$ (18)

The adaptive weight *H* of the SM can be represented as:

$$H(smellIndex(i))=\left\{\begin{array}{c} 1+r.\log\left(\frac{bG-J(i)}{bG-wG}+1\right);\;\\ first\;half\;of\;population\\ 1-r.\log\left(\frac{bG-J(i)}{bG-wG}+1\right);\\ other\;half\;of\;population \end{array}\right.$$ (19)

SmellIndex=sort(J) (20)

Eqs (19) and (20) show that bG is similar to the best fitness solution obtained at the present location, whereas wG is similar to the worst fitness solution obtained at the most recent position. The Smell Index is a sequential representation of the sorted fitness values.

## 3. System modelling

**Fig 2:**
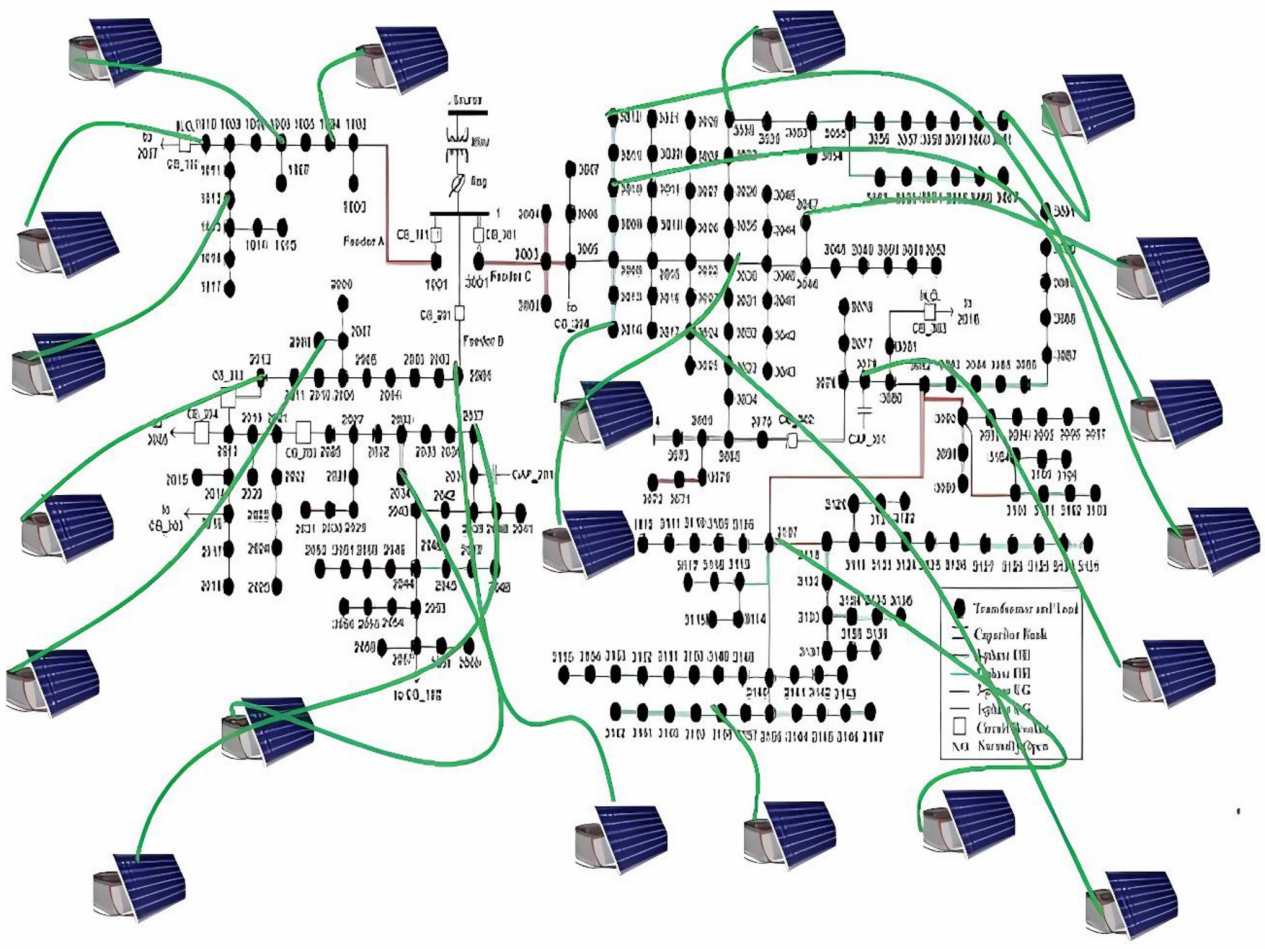
IEEE 240 Bus network.

**Fig 3:**
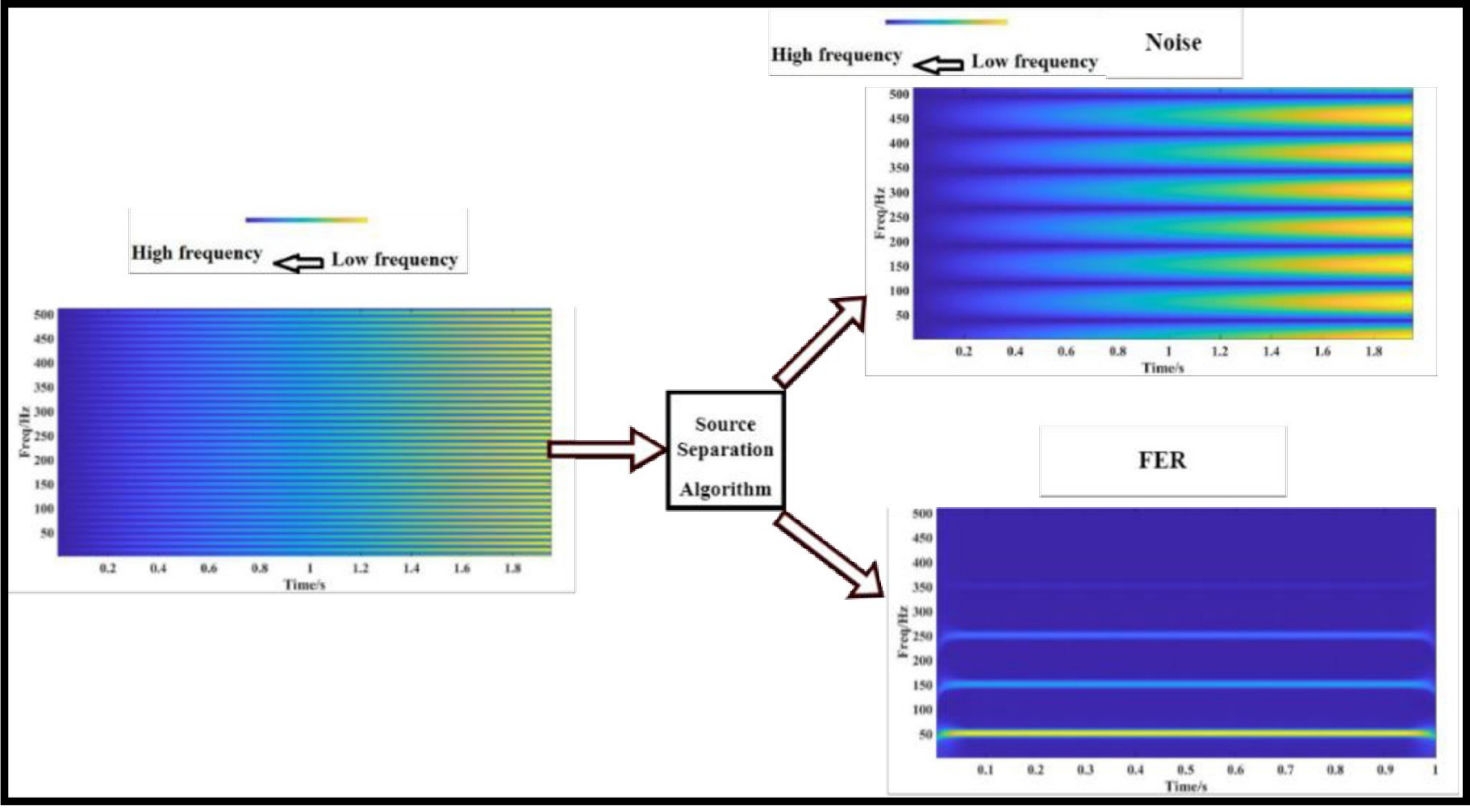
Visualizing the effects of source separation, narrow-band sliding-window spectrograms.

The 240-node distribution system is located in the Midwest of the USA [31]. Three feeders are present in this system, as depicted in Fig 2., 17 nodes on Feeder A, 60 nodes on Feeder B, and 162 nodes on Feeder C receive power from a 69 kV substation. The major length of this distribution system is 23 miles. It supplies electricity to more than 1100 people. Transformers are used for secondary supply clients. Real direct data on power consumption (in kW) is available through SMs that have been put in at different locations based on SMA. There are two capacitor banks at nodes in the 240-bus system. At the substation, there is one on-load tap changer at nodes 2038 and 3079. Installing PV systems at various locations modifies this test system. As depicted in Fig 2, there are a total of 20 PV systems are installed in the 240-node unbalanced distribution system. The location of PVs is shown in Fig 2. The PV system’s size is selected as 40 kVA, even if this PV system sizes and locations are selected on a random basis using empirical data. Voltage readings are taken throughout the grid at various points. In this study, EFs and other transients were generated at 240 bus distribution networks at the bus no 1010, 1015, 2019, 2031, 3022, and 3035. Bus no.1037, 1014, 1005, 1003, 1011, 1015, 2012, 2019, 2030, 2024, 2021, 2032, 2042, 2053, 2066, 2041, 2031, 3034, 3005, 3013, 3011, 3027, 3030, 3042, 3049, 3094 and 3067 are the best locations for the monitoring points which are found by using the Slime Mould optimization technique. As per equation (7–12) the noise is reduced from the collected voltage signal which is represented in the Fig 3.

## 4. Results and discussions

To detect the EFs fault different capacitor bank switching, and feeder energization at various inception angles and locations are simulated to validate the suggested algorithm. Simulations have been carried out with both balanced and unbalanced loads as well as power electronic loads. As devices with this sampling frequency are commercially accessible. The simulation of the signal was executed at a sampling frequency of 2048 Hz and with a time step of 0.1 s. The computation of Q, R, and S was finished one cycle following the fault’s occurrence.

**Fig 4:**
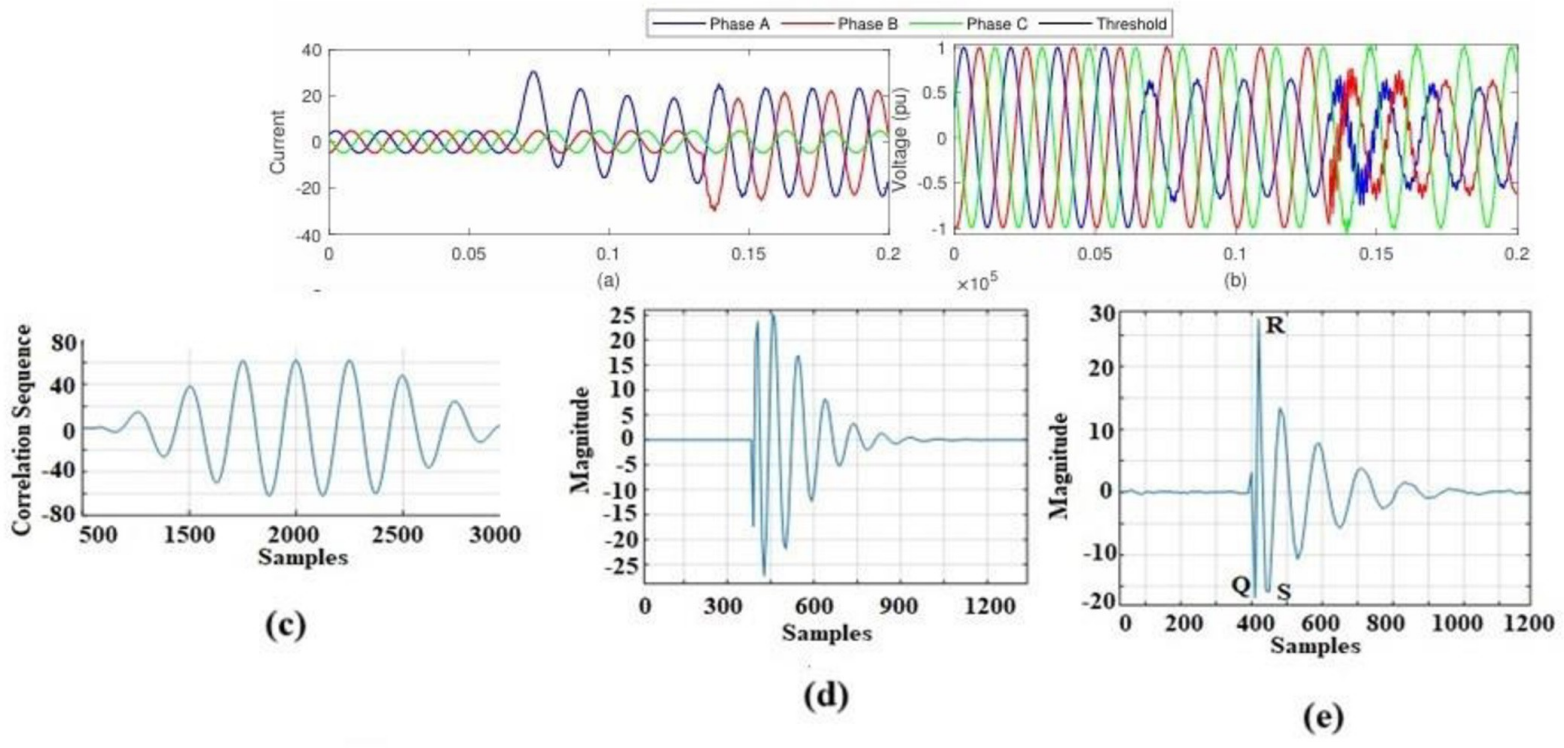
The evolution of feature extraction for ABG failure event detection at bus 10: (a) Bus 10 current signal (b) Bus 10 voltage signal (c) Phase B correlation with pure, and (d) Signal correlation after MOPD operation and filter operation.

The description is divided into two parts, Fig 4(A) and 4(B), each focusing on different aspects of the fault incidents. Fig 4(A): current changes during faults at Phase-A Current, which indicates that the current in Phase-A has increased at the time of an AG fault incidence at the 4^th^ cycle. The increase in current suggests a change in the electrical characteristics, likely due to the fault. Phase-B Current states that the current in Phase-B has also increased at the 8^th^ cycle when the AG fault has evolved into the ABG fault. This change in Phase-B current may be a consequence of the fault propagation and evolution.

**Fig 5:**
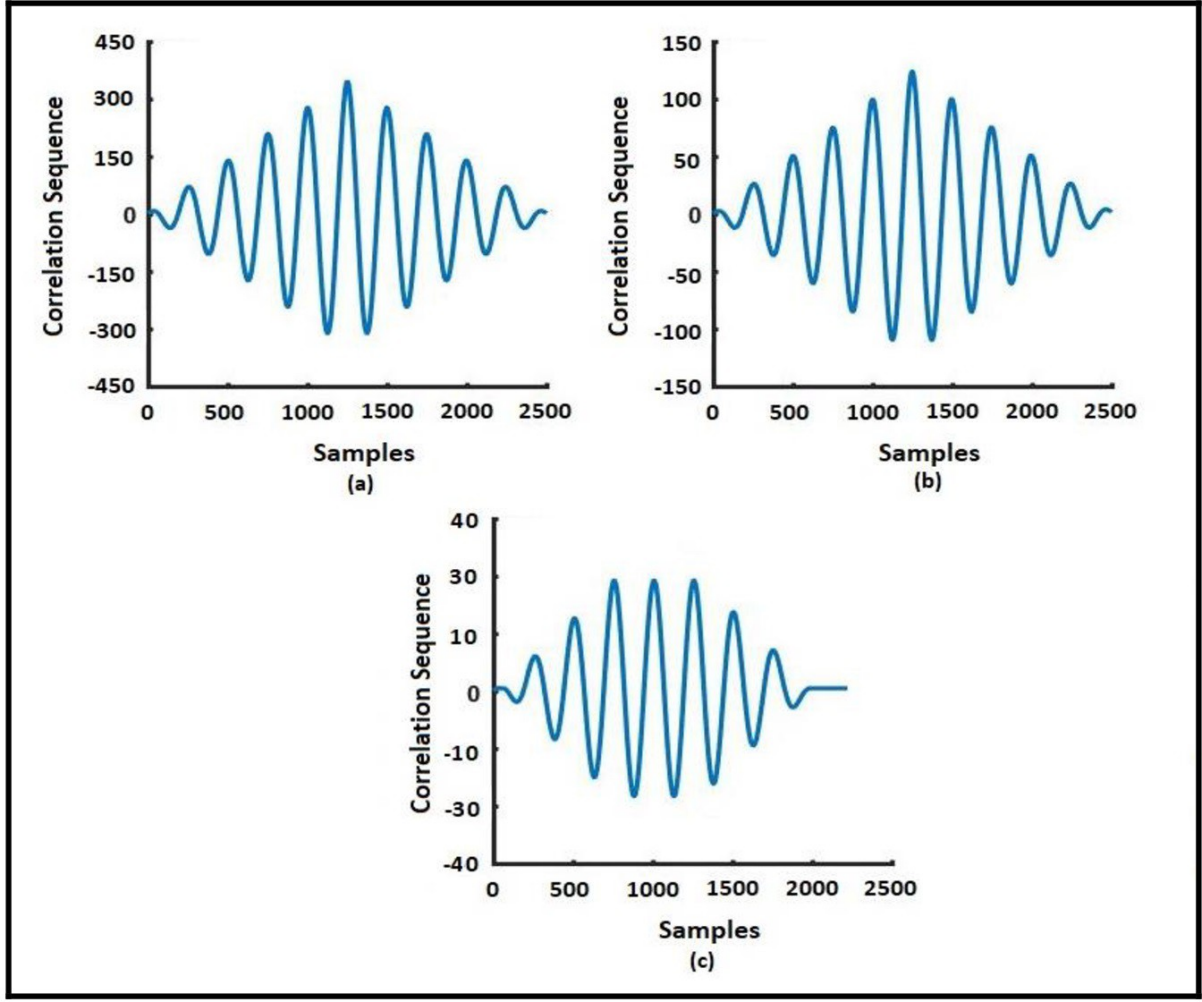
Cross Correlogram: (a)EFs (AG to ABCG), (b) (AG to ABG) and (c) normal short circuit fault (AG).

Fig 4(B) represents voltage changes during Faults at Phase A. It illustrates a decrease in the voltage of Phase-A at the time of an AG fault incidence at the 4^th^ cycle. The voltage reduction in Phase-A is likely a response to the fault occurrence. Phase-B voltage describes a decrease in the voltage of Phase-B at the 8^th^ cycle when the AG fault has evolved into the ABG fault. Similar to phase A, the decrease in voltage in phase B is associated with fault evolution. At the measuring point, voltage signal correlation has been tested under both normal and various transient conditions like cross-country faults. The cross-correlogram between the two signals becomes the autocorrelation of them if the system is functioning properly. The cross-correlogram produces various waveforms for various types of transients. Extraction of features for EFs detection at bus 10 (a) voltage signal during EVs (b) correlation with pure signal, (c) signal after Correlation and MOPD operation, and (d) MOPD operation of normalized signal after filtering. As such, a relocation of a 100-sample-wide window is used, with each ten-sample increment representing one complete cycle which is shown in Fig 5. Since there are two periodic switching fingerprints in two consecutive windows that don’t overlap, we can get rid of them by subtracting the matching data from both windows. A technique based on CC is used to guarantee that the difference is small or zero under typical conditions. This is done by calculating the cross-correlation of the windows at varying delays. The windows’ similarity is at its highest when the estimated cross-value correlation is maximized, and the windows’ length is recalculated after taking the appropriate latency into account. This method guarantees that the subtraction of similar samples yields the correct result.

### A). EF detection technique

**Fig 6:**
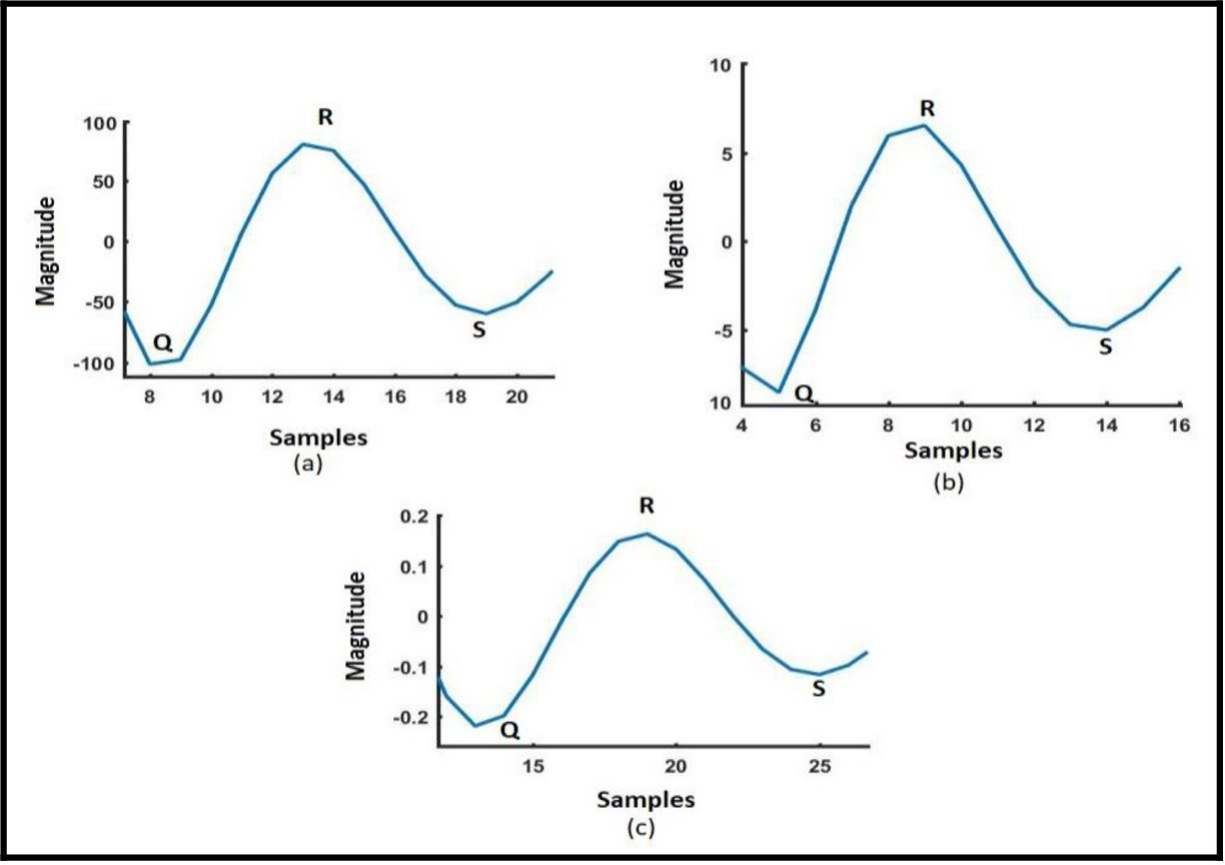
A cross-correlogram that shows the Q, R, and S points after MODP and filter operations. a. EFs (AG to ABG); b. CCAG; and C. Normal Short Circuit Fault (AG).

The method for extracting the correlogram’s essential features for EFs detection is shown in Fig 6. A sinusoidal signal with AG faults in phase A, evolved into ABCG Fault at bus 1020 and monitored from bus 1015 is shown in Fig 6(A). Fig 6 displays the CC between the pure signal and the signal with EFs-ABCG (b). There are three positive maximum values, as shown in Fig 6(A). The signal needs to be further analyzed to determine the highest peak among the three sites. As a result, the maximum peak is determined using the difference operation approach. In Fig 6, the signal following MOPD action which is shown in Fig 6(C). It has been observed that during EFs the magnitude of QRS is high in the range of 100. But during cross-country faults its range is different and under normal short circuits, the value of QRS is very low. The value of QRS will help to discriminate the different types of faults.

### B). Changing the fault inception angle

**Fig 7:**
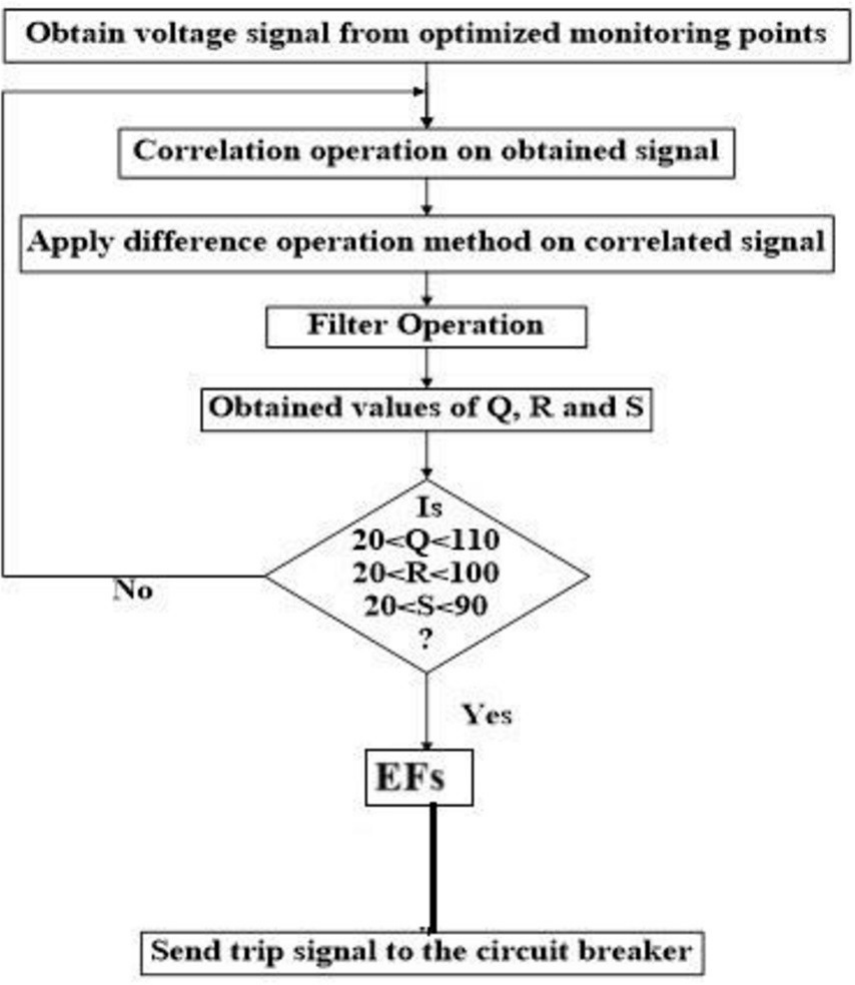
Flowchart of EF detection technique.

To simulate EFs using the recommended technique, three inception angles of 0 degrees, 45 degrees, and 90 degrees have been taken into consideration under three distinct situations, as shown in Table 1. It has been noticed that the values of Q, R, and S all fall within a particular range and that the ranges of Q, R, and S fluctuate regardless of the transient. This is something that has been seen. The ranges of Q, R, and S values for a variety of examples are presented in Table 1, and the flow chart for the EFs detection procedure is shown in Fig 7, respectively. According to Table 1, the Q, R, and S values of the faulty phases are calculated for EFs, CC single line to ground faults, and normal short circuit faults. The investigation was conducted for the three different inception angles of 0°, 45°, and 90°.

### C). Location of EF

In spite of their best efforts, protection experts are still having trouble locating the source of evolving faults. The bulk of the researchers have previously proposed algorithms that are capable of detecting separate faults; however, these techniques are not suitable for actual failures that occur across many countries in real-time. A collection of feeder data is necessary to pinpoint the precise location of the fault so that the approaches that have been developed may be utilized. However, the search field area may be greatly decreased by examining the Q, R, and S values of connected signals of healthy and defective phases from a variety of monitoring stations. This is the case even if the research that is being presented is unable to pinpoint the precise position of the EFs point specifically. The search area will thus become substantially more limited as a consequence of this. Using the approach that was provided, the values of Q, R, and S will all be at their lowest possible levels if the correlation is performed between the voltages of the three phases under normal conditions. This is because the voltages in this situation are at their usual values. If an EF occurs in a bus, the voltages of the buses that are near the bus are most strongly affected. The voltage of the bus rises as the distance from the fault grows, and it reaches its greatest point when the bus is in the closest proximity to the defective bus. Consequently, Q, R, and S have high values close to the bus that was destroyed, and their values increase as the monitoring stations travel further away from the bus. It has been seen from a variety of optimal monitoring stations and the recommended technique has been tested at many different fault locations. Table 2 provides a summary of the data that is linked with this. A single line-to-ground fault that was located on bus 1011, bus 10133, and bus 1027 has been transformed into a double line-to-ground fault, and the voltage signal is being monitored (Fig 2). When the cross-country fault develops at bus 1012 at a 45° inception angle, the magnitudes of Q, R, and S are shown to be the least at the nearest monitored site, which is bus 1009 and 1015, as shown in Table 2. At the three nearest monitoring stations, 1014, 1005, and 1011, the highest Q, R, and S values for the problem at bus 3 at an inception angle of 45 degrees were calculated. These monitors are all at the same distance from the defective bus and produce findings that are comparable to one another. These two places are located at the same distance apart. Assuming once more that there is a problem with bus number 1027, the neighboring bus will have high values of Q, R, and S because it is the optimum monitoring point that is closest to the site of the problem. This is because the neighboring bus is the one that is closest to the problem. The monitoring point that is positioned closest to the defective bus will offer the highest values for Q, R, and S, whilst the monitoring point that is located the farthest away from the bus will provide the lowest values for Q, R, and S. This is so long as all of the monitoring points are observed for each of the faults listed in Table 2. If each monitoring point is noticed, then this will be the case. The reason for this is that the bus that is physically placed in the shortest distance from the bus that was damaged has a high voltage, which suggests that the location of the faults in the electric vehicles is the bus that is to blame. To get the most cost-effective solution for the essential data, it is necessary to strike a balance between the desired level of precision and the greatest amount of money that can be spent on metering.

**Table 2:** Values of Q, R, and S values for different fault conditions.

Faulted Phase/correlated phase	Correlation of	EF condition 1			EF condition 2			EF condition 3		
		Q	R	S	Q	R	S	Q	R	S
B &C correlated with phase A	A	4.01	3.1	3.1	0.04	0.03	0.03	4	3	3.02
	B	82	97	87	0.87	0.81	0.9	0.95	81	82
	C	81	95	87	0.87	0.84	0.9	0.96	82	82

### D). Influence of noise

Noise is always present in real-time systems, and it is present in both the voltage and current signals. Noise or interference is the term used to describe unwanted electrical impulses that interfere with or distort a signal that was intended to be received. Because of this, the efficiency of the recommended method for detecting HIF across several countries has also been tested in noisy situations. A normal probability distribution may be seen in the power system noise that is present throughout the whole recorded signal. Here is the equation for the signal-to-noise ratio (SNR), which indicates that this noise is:

$$SNR_{dB}=20.\log_{10}\left(\frac{A_{signal}}{A_{noise}}\right)$$ (23)

**Fig 8:**
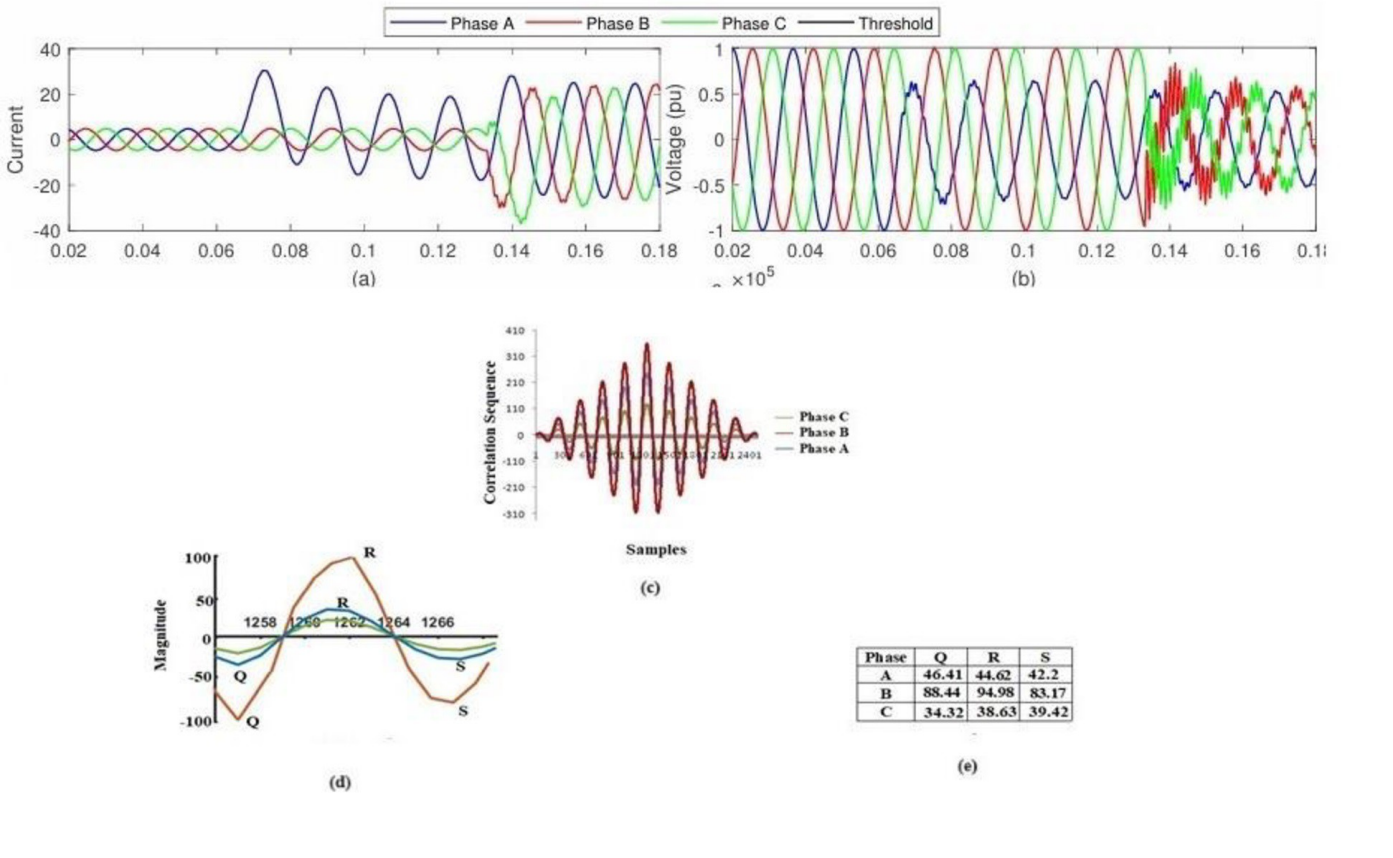
EF detection while noise is present: (a) The signal strength at the monitoring location (b) The voltage signal at the monitoring site; (c) A correlation diagram showing the dysfunctional phase B with the healthy phases A and C; (d) The Q, R, and S values for the three phases; and (e) The voltage signal at the monitoring point.

To limit the influence of random noise that is not associated with the signal and to provide trustworthy findings in a noisy environment, the proposed research makes use of an approach that is based on cross-correlation. Fig 8 illustrates a succession of voltage waveforms that were produced by EF under circumstances where noise was present at many different locations. It has been determined through study that the noise effect does not have any influence on the EF detection standard, and the approach that was presented has been incorporated into the system. Fig 8 depicts the cross-correlogram of the two signals, which allows for the detection of the cross-country EF in the presence of random noise that is not correlated with the current signal.

**Fig 9:**
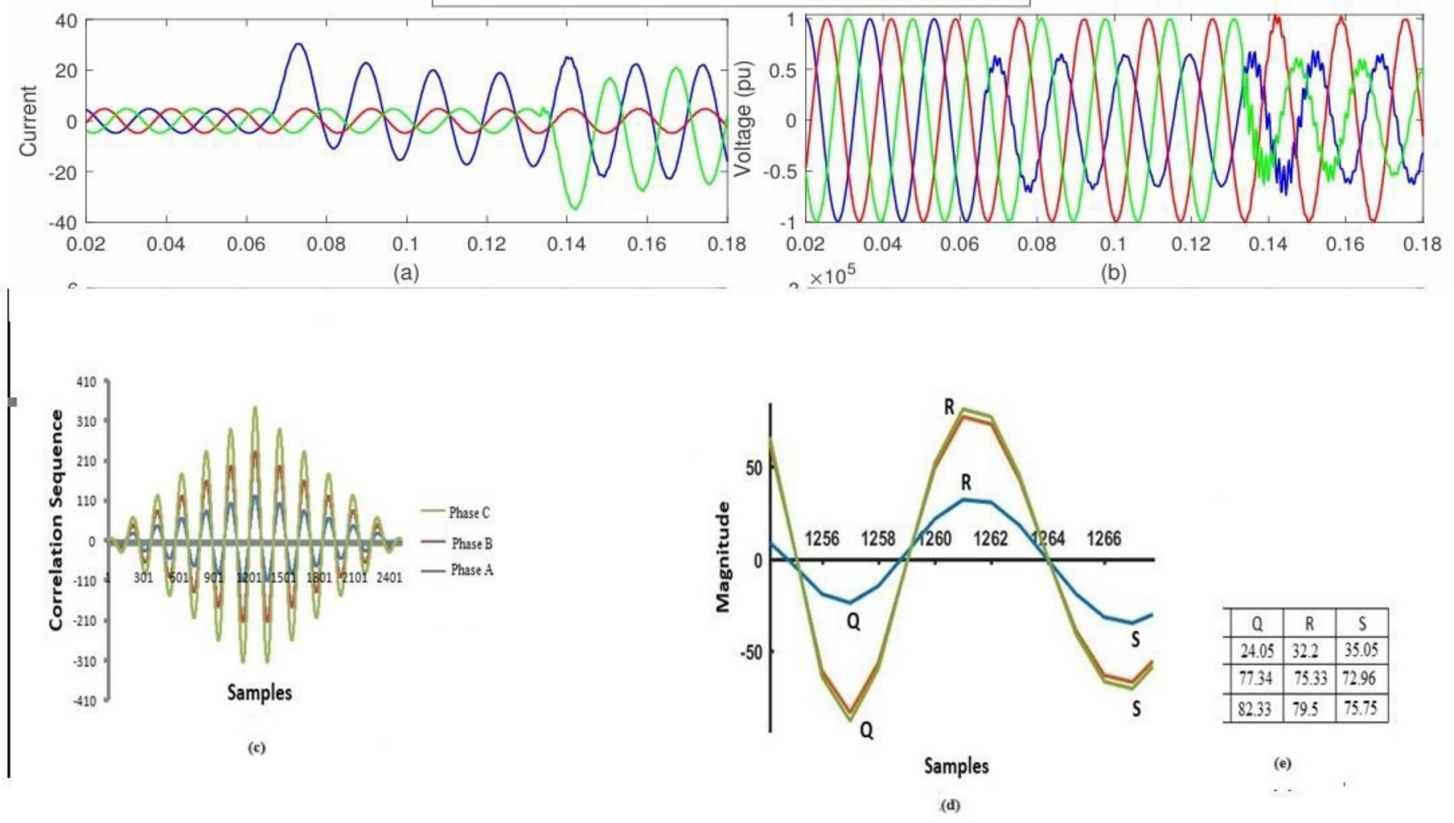
The identification of EFs under Distributed Generation: a) The current signal that is present during the fault; b) The voltage signal at the monitoring point; c) The correlation of the faulty phase B and the healthy phases A and C; d) The Q, R, and S values of the faulty phase B and the healthy phases A and C; and e) The values of Q, R, and S that were obtained for all three phases.

Since DG has the potential to supplant traditional energy sources in the power market, it is a hot subject right now. Simulations have been conducted taking into account the system model with distributed generation, following the current trend of grid-connected distributed generation technologies. The suggested approach involves linking a wind farm to the IEEE 240 bus system through bus 10. A Δ/Yn transformer with a rating of 1.75 MVA, 575V/ 12.66kV is linked to the six 1.5MW wind turbines that make up the wind farm. An AC/DC/AC IGBT-based pulse width modulator (PWM) converter and a wound rotor induction generator (DFIG) are the components of the wind turbines. EF has been generated at nodes 3, 10, and 27 via distributed generation and is being observed from all the optimum monitoring points. It is applicable to all inception angles, fault resistances, and other transients. When EF happens in phase B at bus 3, it evolves into three phases to ground faults. Fig 9(A)–9(D) show the voltage signal at bus 32, the correlogram of the healthy and faulty voltages, the location of Q, R, and S on the correlogram, and the corresponding values of Q, R, and S, respectively. According to the results, the suggested technique is better than the alternatives, and the values of Q, R, and S all fall within the range shown in Fig 9.

### E). EF detection influence of power electronics load

**Fig 10:**
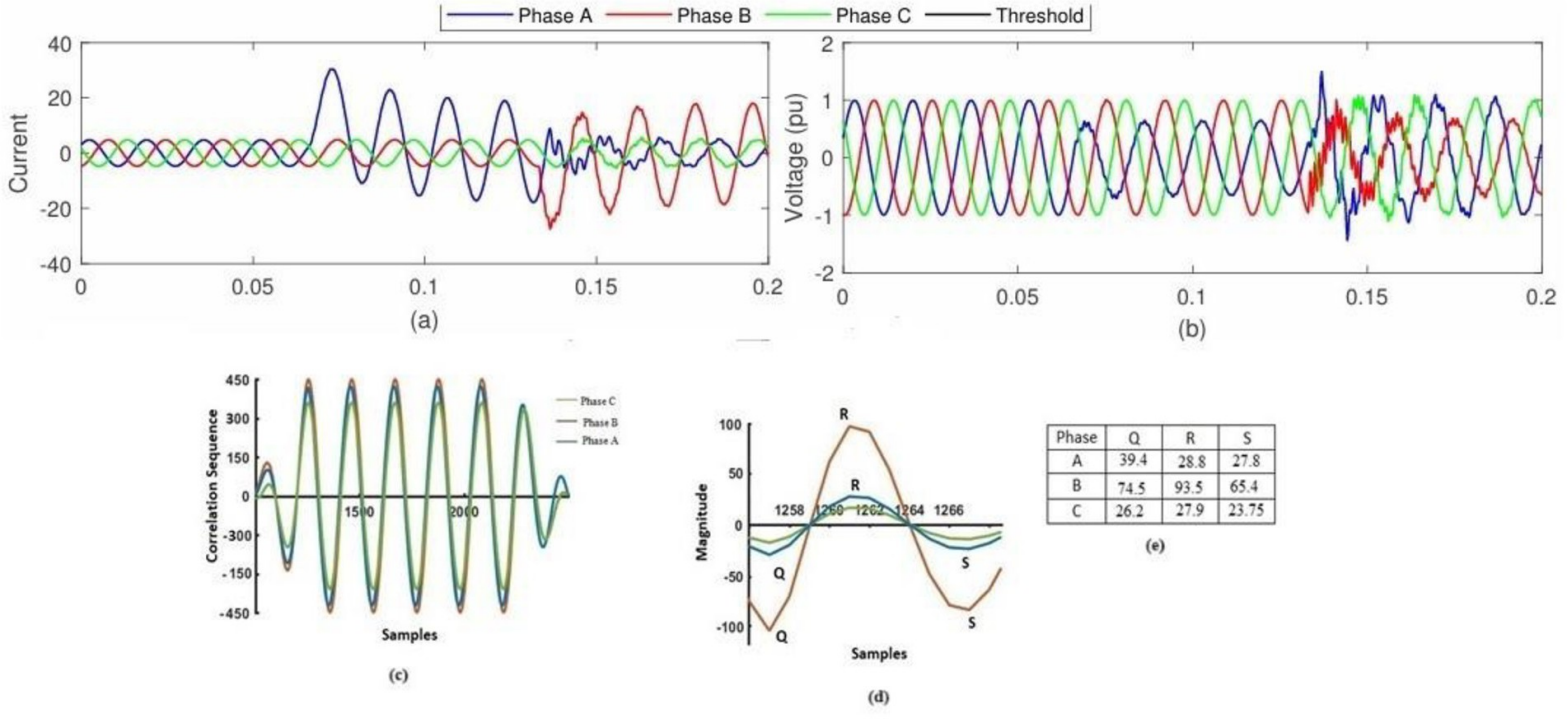
The detection of EF in the presence of non-linear loads that are interfaced with power electronics:(a) The current signal seen at the monitoring point (b) the voltage signal observed at the monitoring point (c) A correlation is drawn between the faulty phase B and the healthy phases A and C; (d) the Q, R, and S values of the faulty phase B and the healthy phases A and C; and (e) the values of Q, R, and S that were obtained for all three phases.

Power electronics interfaced nonlinear loads, such as time-varying harmonics in DNs, are now commonly used by consumers in both the residential and industrial sectors. To evaluate the effectiveness of the suggested approach, it was put through a series of tests using a non-linear load. The design of the non-linear load is accomplished by the utilization of a 6-pulse converter bridge. This bridge is connected at various locations along a 240-bus distribution network, and it supplies a direct current load of 650 kW at 3.6 kV. As a result of the existence of converter load at many different places, cross-country EF and other types of transients have been generated, and the waveforms of the voltage are shown in Fig 10. The suggested method is incorporated into the system, and the results of the investigation show that the converter loads do not have any impact on the EF detection standard. As a result, the suggested method may identify the cross-country EF when non-linear power electronics loads are interfaced. From Fig 10 it is clear that EF occurs at phases A and B (correlated with phase C).

A condition of uneven loading has also been used to evaluate the suggested cross-country EF detection technique. The proposed network is already unbalanced. Cross-country EF has been constructed for buses 1002, 1004, and 1027 and monitored from optimal monitoring bus sites for detection of EF under unbalanced loading. Cross-correlation between the unbalanced voltage at bus 2 under normal conditions and the unbalanced voltage with EF has been performed. The cross correlogram of the voltage waveform, whose values are comparable to those observed during a balanced load situation, has been used to evaluate the values of Q, R, and S. As a result, the unbalanced loading condition does not affect the suggested technique. Then, EF has been developed in phases A and B of bus 1027 and is being watched from bus 1024 as shown in Fig 10. This establishes the algorithm’s suitability for cross-country EF detection during unbalanced loading.

Several methods for locating cross-country EF have previously been covered in this article. Using a real-time simulator, the performance of the suggested technique under dispersed generation and power electronic interfaced non-linear loads has been investigated For the suggested approach to identify cross-country EF syndrome, a three-phase voltage signal is needed at any one monitored location.

### F). Comparative study

A comparative study of EFs in a DN involves analysing and comparing the characteristics, impacts, and responses to faults as they develop or evolve over time. Here are key aspects to consider in such a study:

**Fault types:**
  Identify and classify different types of faults in a DN, such as short circuits, open circuits, ground faults, and transient faults.
  Understand the characteristics of each fault type, including their initiation, development, and possible outcomes.
**Fault detection and monitoring**
  Investigate methods and technologies for detecting EFs in DNs.
  Compare the effectiveness of different fault detection techniques, such as current and voltage measurements, relays, and smart grid technologies.
**Impact on system performance:**
  Analyze the impact of EFs on the overall performance of the DN.
  Evaluate how faults affect system reliability, power quality, and operational efficiency.
**Response and mitigation strategies:**
  Examine various response and mitigation strategies for EFs, including automatic reconfiguration, fault isolation, and adaptive protection schemes.
  Compare the effectiveness of traditional protection methods with emerging smart grid technologies.
**Data analysis and machine learning:**
  Explore the use of data analytics and machine learning techniques for fault prediction and classification.
  Compare the performance of different algorithms in predicting the evolution of faults based on historical data.
**Communication and control systems:**
  Investigate the role of communication and control systems in responding to EFs.
  Compare the performance of different communication protocols and control strategies in minimizing the impact of faults.
**Resilience and recovery:**
  Assess the resilience of the distribution system to evolving faults and its ability to recover quickly.
  Compare recovery times and methods for different fault scenarios.
**Case studies:**
  Analyze real-world case studies of evolving faults in distribution systems to provide practical insights.
  Compare the outcomes of different fault scenarios in terms of downtime, economic losses, and customer impacts.
**Regulatory and standards compliance:**
  Consider regulatory requirements and standards related to fault detection, response, and system reliability.
  Compare how different distribution systems comply with these regulations and standards.
**Future trends and technologies:**
  Explore emerging trends and technologies in distribution systems, such as the integration of renewable energy sources, microgrids, and advanced sensing technologies.
  By conducting a comprehensive comparative study in these areas, you can gain insights into the evolving nature of faults in distribution systems and identify optimal strategies for fault detection, response, and mitigation.

## 5. Conclusions

The research described presents a novel approach for detecting EFs in distribution networks using correlation-based methods, focusing on voltage signals at a single monitoring point. Here’s a breakdown of the method and its advantages:

- Correlation-based detection: The method relies on analysing voltage signals at a single monitoring point using cross-correlation operations. This simplifies the detection process and reduces the computational burden compared to more complex techniques. By focusing on voltage signals, the method can effectively identify evolving faults even under various circumstances such as capacitor bank switching, load switching, feeder energization, and unbalanced loading.
- Feature extraction: Only three features from the cross correlogram of voltage signals are used to identify the faulty phase during evolving fault conditions. This demonstrates a streamlined approach to feature extraction, enhancing computational efficiency.
- Noise reduction: The cross-correlation approach helps reduce the impact of random uncorrelated noise from the signal, improving the robustness of the fault detection method.
- Optimization technique: SMA is utilized to determine the optimal locations of monitoring points in the network for fault detection. This highlights a proactive approach to optimizing monitoring infrastructure, potentially reducing costs and improving system effectiveness.
- Real-time verification: The method is validated in a real-time platform, demonstrating its efficiency and practical applicability.
- Simplicity and independence: The method’s simplicity is notable as it doesn’t require distributed parameters, advanced artificial intelligence techniques, or time synchronization with monitoring devices. This makes it more accessible and easier to implement in distribution networks.

Overall, the described approach offers a promising solution for detecting EFs in DNs, leveraging correlation-based methods, streamlined feature extraction, noise reduction, optimization techniques, and real-time validation. Its simplicity and effectiveness make it a valuable addition to fault-detection strategies for distribution systems.
